# Urinary Netrin-1: A New Biomarker for the Early Diagnosis of Renal Damage in Obese Children

**DOI:** 10.4274/jcrpe.2828

**Published:** 2016-09-01

**Authors:** Duygu Övünç Hacıhamdioğlu, Bülent Hacıhamdioğlu, Demet Altun, Tuba Müftüoğlu, Ferhan Karademir, Selami Süleymanoğlu

**Affiliations:** 1 Gülhane Military Medical Academy, Haydarpaşa Training and Research Hospital, Clinic of Child Health and Diseases, İstanbul, Turkey; 2 Etimesgut Military Hospital, Clinic of Pediatrics, Ankara, Turkey; 3 Gülhane Military Medical Academy, Haydarpaşa Training and Research Hospital, Clinic of Biochemistry, İstanbul, Turkey

**Keywords:** obesity, insulin resistance, tubular dysfunction, netrin-1, children

## Abstract

**Objective:**

Urinary netrin-1 is a new marker to demonstrate early tubular damage. The aim of this study was to determine whether urinary netrin-1 is increased in obese children.

**Methods:**

A total of 68 normoalbuminuric and normotensive obese patients and 65 controls were included in the study. Urine samples were collected for assessment of urinary phosphorus, sodium, potassium, creatinine, albumin, and netrin-1. Blood samples were collected for measurements of fasting glucose, insulin, lipid, phosphorus, sodium, potassium, and creatinine levels. Homeostatic model assessment insulin resistance index was calculated.

**Results:**

Gender and age were similar between obese and control groups (12.01±3.03 vs. 11.7±3.2 years, p=0.568 and 33 vs. 35 girls, p=0.543, respectively). Obese patients had significantly higher netrin-1 excretion than the controls (841.68±673.17 vs. 228.94±137.25 pg/mg creatinine, p=0.000). Urinary netrin-1 level was significantly higher in obese subjects with insulin resistance compared to those without insulin resistance (1142±1181 vs. 604.9±589.91 pg/mg creatinine, p=0.001).

**Conclusion:**

In normotensive and normoalbuminuric obese children, urinary netrin-1 level can increase before onset of albuminuria. Urinary netrin-1 excretion appears to be affected predominantly by insulin resistance and hyperinsulinemia. Urinary netrin-1 may be a new biomarker for determining early tubular injury in obese children.

**WHAT IS ALREADY KNOWN ON THIS TOPIC?**

Netrin-1 is a promising early biomarker of tubular kidney injury. The impact of obesity on chronic kidney disease has been well demonstrated.

**WHAT THIS STUDY ADDS?**

Netrin-1 is significantly elevated in obese patients without microalbuminuria. Netrin-1 was higher in obese with insulin resistance than without insulin resistance.

## INTRODUCTION

The impact of obesity on chronic kidney disease (CKD) has been well demonstrated (1,2). The diagnostic test currently used for CKD in clinical practice is increased albumin excretion rates. However, this is a sign of early glomerular damage rather than a marker for susceptibility to it. Also studies assert that tubulointerstitial injury may precede the appearance of glomerulopathy in diabetic nephropathy (3,4). Therefore, identification and validation of tubular injury biomarkers for early diagnosis of kidney injury come into prominence.

The netrin-1 is a conserved family of laminin-related proteins that were originally identified as axonal guidance cues (5). Many studies have demonstrated netrin-1 expression outside the nervous system, including the kidneys (6). Using both in vitro and in vivo systems, netrin-1 was shown to play a role in promoting angiogenesis, cell migration, tissue morphogenesis, and in regulation of inflammation (7,8). Netrin-1 protein expression has been localized to endothelial cells in the normal kidney, and very little protein expression is seen from tubular epithelial cells (9). After injury, netrin-1 protein expression appears in proximal tubular epithelial cells but, at the same time, the expression in vascular endothelial cells is down regulated. Since netrin-1 is a secreted protein in urine, this led to the discovery of netrin-1 as an early diagnostic biomarker of kidney injury (8,9). It was found that netrin-1 is a secreted protein highly induced after acute and chronic kidney injury and excreted in urine in both mice and humans (10,11).

Reports in the literature show that tubular changes such as hypertrophy, reduced ion transport, and thickening of the basement membrane are already apparent before the onset of proteinuria in early diabetic nephropathy (3,4). In a recent study, urinary netrin-1 levels were significantly increased in normoalbuminuric diabetic adult patients when compared to healthy controls and still further elevated in patients with microalbuminuria and overt nephropathy (12). We have hypothesized that urinary netrin-1 excretion was higher in obese children than healthy children. We therefore compared the netrin-1 levels between normoalbuminuric/normotensive obese children and controls. Furthermore, we examined the risk factors affecting the level of urinary netrin-1.

## METHODS

This cross-sectional study examined two groups (obese patients and healthy controls) attending the pediatric endocrinology outpatient clinic of our hospital. All patients provided written informed consent, and our ethics committee approved the study protocol. Financial support was received from the Institute’s Epidemiological Committee.

Obesity was defined as a body mass index (BMI) ≥95^th^ percentile for age and sex. Normal weight was defined as BMI <85^th^ percentile for age and sex (13). Normotension was defined as a systolic or diastolic blood pressure <90^th^ percentile according to age, sex, and height on at least three occasions. Hypertension was defined as systolic or diastolic blood pressure ≥95^th^ percentile according to age, sex, and height on at least three occasions (14). We used the United States National Heart, Lung, and Blood Institute (NHLBI) definition for dyslipidemia, abnormal values being >95^th^ percentile, except for high density lipoprotein, for which an abnormal value is less than 10^th^ percentile (15). Insulin resistance was defined according to the homeostatic model assessment of insulin resistance (HOMA-IR). HOMA-IR was estimated from fasting plasma measurements [insulin (μU/L) X glucose (mg/dL)/405]. Insulin resistance criteria were HOMA-IR >2.67 for prepubertal boys, >2.22 for prepubertal girls, >5.22 for adolescent boys, and >3.82 for adolescent girls (16). Microalbuminuria was defined according to the urine albumin-to-creatinine ratio. A value of >30 mg/g for creatinine suggests moderately increased albumin excretion (17).

Each child underwent a complete physical examination and anthropometric measurements, including pubertal staging according to the Tanner criteria (18,19). Height and weight were measured in postabsorptive conditions and with an empty bladder. Height was measured to the nearest 0.5 cm on a standard height board, and weight was determined to the nearest 0.1 kg on a standard physician’s beam scale with the subject dressed only in light underwear and no shoes. To compare BMI across different ages and between genders, BMI standard deviation score (SDS) was calculated.

Patients with the following were excluded from the control group: obesity; chronic disease; family history of stroke, diabetes or dyslipidemia, use of any medications during the study period or in the preceding 6 weeks, and existing or previous infections in the preceding 6 weeks according to the patients’ clinical history and physical examination. Participants in the study were selected from among healthy children who presented to our pediatric outpatient clinic for evaluation of fitness for sportive activities.

The inclusion criteria for the study group consisted of being obese, normoalbuminuric, and normotensive. Exclusion criteria were hypertension, diabetes mellitus, microalbuminuria, syndromic obesity (Prader-Willi, Laurence-Moon Biedl syndrome, etc.), endocrine disease such as Cushing’s syndrome or hypothyroidism, systemic disease including liver disease, malignancy, as well as existing or previous infection and drug use.

Fresh first morning urine samples were collected for determination of urinary phosphorus, sodium, potassium, creatinine, and netrin-1 levels. Normoalbuminuria was confirmed by two different first morning urine samples. Blood samples were also taken for determination of fasting glucose, insulin, total cholesterol, low-density lipoprotein (LDL), triglyceride, phosphorus, sodium, potassium, and creatinine levels. All determinations were done within 4 hours of sample collection, except for netrin-1, where urine samples were centrifuged (3,000 rpm for 20 min) and stored at -80 °C until measurement. Estimated glomerular filtration rate (eGFR) was calculated according to the Schwartz formula (20). The fractional excretion of sodium (FE_Na_), fractional excretion of potassium (FE_K_), and tubular phosphate reabsorption (TPR) rate were calculated using standard formulas.

Serum and urinary phosphorus, creatinine, total cholesterol, LDL, and triglycerides were measured by the colorimetric method; serum and urinary sodium and potassium were determined by indirect method in ISE module with Abbott Architect autoanalyzer C16000 (Illinois, USA) using home-made reagents. Serum creatinine was measured using the compensated Jaffe method. Urinary microalbumin was estimated using the nephelometric method with Siemens Nephelometry analyzer (Erlangen, Germany).

Urinary netrin-1 was measured using the enzyme-linked immunosorbent assay (ELISA) method (Sunred, Shanghai, China). Intra-assay and inter-assay precision were <9% and <11%, respectively. All assays were performed in duplicate. Urinary netrin-1 excretion is expressed in picograms (pg) per mg of creatinine.

All statistical calculations were performed using SPSS for Windows 15.0 (SPSS, Chicago, IL, USA). Comparison between two groups was performed using Mann-Whitney U-test for non-normally distributed parameters and student’s t-test for parameters showing normal distribution. Comparison between subgroups was performed with Kruskal-Wallis tests. The means in more than two groups were compared using one-way ANOVA. Where the p-value was significant, pairwise comparisons were done with post-hoc Bonferroni test. Statistical significance was accepted as p<0.05. The parameters are expressed as mean values and standard deviations. Spearman correlation coefficient was used for correlation analysis. After adjustment for age and gender, we assessed the associations between netrin-1 and other relevant parameters by partial correlation analysis.

## RESULTS

A total of 62 obese patients and 64 control subjects participated in this study. The demographic characteristics and biochemical parameters of the two groups are shown in Table 1. There were no differences between the groups for age, gender, serum creatinine, eGFR, urine albumin-to-creatinine ratio (p>0.05). However, obese patients had significantly higher netrin-1 excretion when compared to controls [477.6 (240.3-864.5) vs. 240.9 (123.5-450.2) pg/mg creatinine, respectively, p=0.00064].

In the obese group, 11 subjects (17.7%) had dyslipidemia. When we compared the dyslipidemic and normolipidemic subjects, there were no differences in age, gender, BMI SDS, HOMA-IR, urine albumin-to-creatinine ratio, eGFR, FE_Na_, FE_K_, TPR, and urinary netrin-1.

We divided the obese subjects according to insulin resistance (Table 2). There were no differences between patients with and without insulin resistance for age, gender, BMI SDS, eGFR, urine albumin-to-creatinine ratio, FE_Na_, FE_K_, TPR, total cholesterol, and LDL. However, urinary netrin-1 was significantly higher in patients with insulin resistance than patients without insulin resistance [645.6 (480.4-1012.5) vs. 431.3 (207.2-682.5) pg/mg creatinine, p=0.005].

In order to evaluate the factors associated with urinary netrin-1, we performed correlation analyses (Table 3). There was no significant correlation between urinary netrin-1 and age, gender, BMI SDS, eGFR, urine albumin-to-creatinine ratio, FE_N_a, FE_K_, TPR, total cholesterol, and LDL. There were positive correlations between urinary netrin-1 and fasting insulin, glucose, and HOMA-IR (r=0.279, p=0.022 vs. r=0.433, p=0.000 vs. r=0.345, p=0.004, respectively). Partial correlation analysis was performed considering age and gender (Table 4). Even after considering age and gender, the results did not change.

## DISCUSSION

Baseline BMI has been suggested as an independent predictor of CKD progression (2). Metabolic syndrome, a major consequence of obesity, also seems to be an independent risk factor for end-stage renal disease (ESRD) (1). For this reason, investigations for a diagnostic test to detect early renal damage in clinical practice have increased. This present study has demonstrated that urinary netrin-1, which seems to be affected by tubular injury, is significantly elevated in obese patients without microalbuminuria when compared to controls. These results may suggest that tubular changes may already be apparent even before the glomerular injury process has begun in obese children.

Microalbuminuria currently used for CKD has significant limitations. The presence of microalbuminuria by itself may not be an adequate indicator for disease progression because of the observation that a number of type 1 diabetes patients revert to normoalbuminuria without treatment (21). Microalbuminuria is suggested to be a sign of early glomerular damage. The occurrence of microalbuminuria in type 1 diabetes can already be associated with diabetic nephropathy lesions comparable to the ones found in overt diabetic nephropathy (21). Therefore, identification and validation of biomarkers for early diagnosis of kidney injury may help develop effective treatment for kidney disease. For this purpose, tubular injury biomarkers such as kidney injury molecule-1 (KIM-1), N-acetyl-β-D-glucosaminidase (NAG), and neutrophil gelatinase-associated lipocalin (NGAL) were investigated in patients with obesity (22). Our study revealed that obese patients had higher urinary NAG and KIM-1 values compared to controls, but that there was no difference in urinary NGAL (22). In another study, it was demonstrated that obese children and adolescents had reduced nitric oxide (NO) levels and increased urinary isoprostanes when compared to normal weight controls (23). We found that urinary netrin-1 is significantly elevated in obese patients without microalbuminuria when compared to controls. However, there is a need for prospective studies in this regard.

Recent evidence supports the hypothesis that reduced insulin sensitivity and hyperinsulinemia are among the most important factors leading to renal injury (24). In a recent study, NAG and KIM-1 were not different in obese patients when checked for impaired glucose tolerance and insulin resistance (23). In another study, reduced NO levels, increased urinary isoprostanes, and blood pressure measurements were all found to be related to insulin resistance (23). Furthermore, we found that urinary netrin-1 level was higher in normotensive obese subjects with insulin resistance when compared to those without insulin resistance. Also, no differences were observed between these subjects for microalbuminuria. In previous studies, it was shown that netrin-1 protein is induced in proximal tubular epithelial cells and excreted in urine during diabetes before microalbuminuria onset both in animal models and in humans (12,25). In our study, the subjects did not have diabetes mellitus or hyperglycemia. Therefore, we believe that the increased netrin-1 in our patients probably was a reflection of the complications of hyperinsulinemia-induced tubular changes. In addition, there were positive correlations only between urinary netrin-1 and fasting glucose, fasting insulin, and HOMA-IR in our study. These data support the idea that hyperinsulinemia and insulin resistance seem to be effective factors for netrin-1 excretion in obese children.

Netrin-1 has a molecular mass of 72 KDa. Therefore, it is unlikely that it is filtered by glomerules under normal conditions. However, netrin-1 may be filtered after renal injury as this is known to cause changes in the filtration barrier (25). In our study, we observed no differences neither in eGFR nor in microalbuminuria between obese and control groups. This finding indicates that increased levels of urinary netrin-1 in obese patients may come from a proximal tubular source. Also, there were no differences in FE_Na_, FE_K_, and TPR values between obese and control groups. It seems that the changes in proximal tubules are detectable by increase in urinary netrin-1 even before the indicators of known proximal tubular function are affected. Thus, the findings of this study confirmed our hypothesis and showed that netrin-1 excretion in urine is increased in obese children. However, prospective studies are required to investigate whether netrin-1 reflects tubular dysfunction due to obesity.

This study has some limitations. The patient number was low. This is a cross-sectional study. Therefore, there is a need for larger prospective studies to confirm the results. We did not check other tubular injury markers like NAG, KIM-1, NGAL. Also, if there was another group with albuminuria, this would strengthen our results. We have tried to overcome the probable false results of ELISA by performing all samples in duplicates.

Obesity has a great influence on ESRD, and it can be either the cause of renal alterations and kidney injury or an aggravating factor in patients with diabetes. Netrin-1 could be a promising early biomarker of kidney injury. For showing tubular damage in obese children, urinary netrin-1 may be a new marker that can increase earlier than conventional markers of renal injury such as microalbuminuria. Insulin resistance and hyperinsulinemia seem to be related to the urinary level of netrin-1. A prospective study is needed to examine the clinical usefulness of urinary netrin-1 excretion in the early tubular injury in obese children.

### Ethics

Ethics Committee Approval: Our ethics committee approved the study protocol, Informed Consent: All patients provided written informed consent.

Peer-review: Externally peer-reviewed.

## Figures and Tables

**Table 1:**
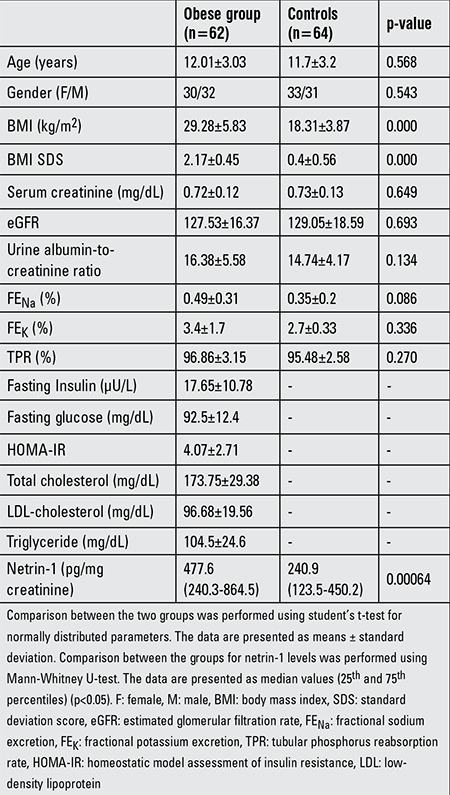
The demographics and biochemical parameters of the obese and control groups

**Table 2:**
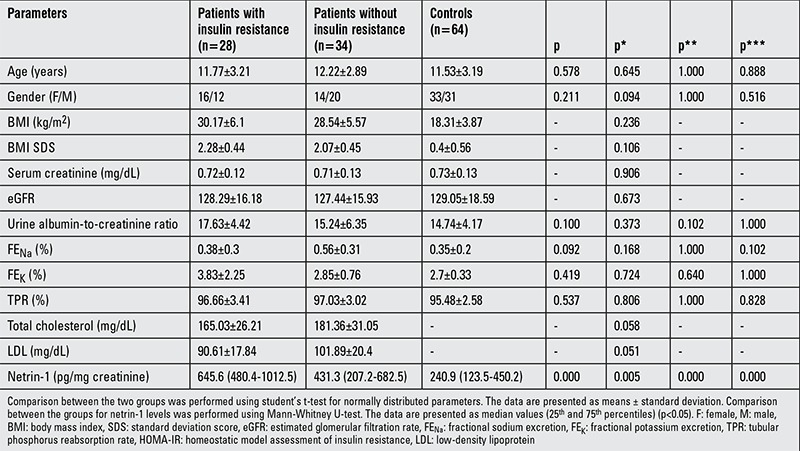
Comparison of patients with insulin resistance with those without insulin resistance and controls

**Table 3:**
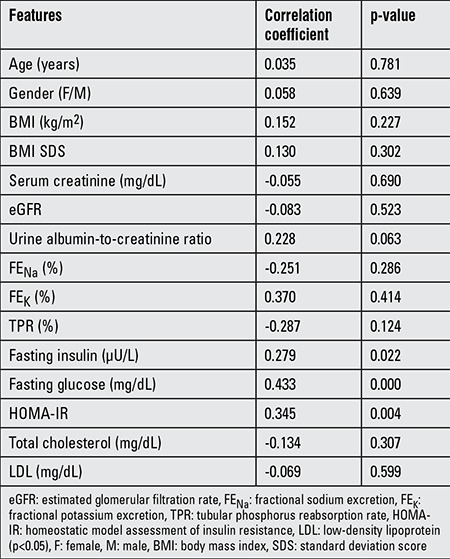
Factors associated with urinary netrin-1 in the obese group

**Table 4:**
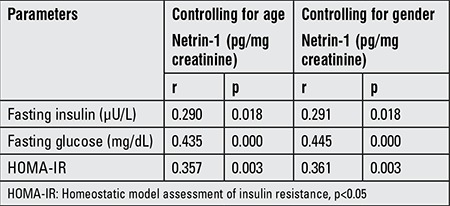
Partial correlation analysis between urinary netrin-1 and fasting insulin, fasting glucose, and homeostatic model assessment of insulin resistance, controlling for age and gender

## References

[1] Wahba IM, Mak RH (2007). Obesity and obesity-initiated metabolic syndrome: mechanistic links to chronic kidney disease. Clin J Am Soc Nephrol.

[2] Wang Y, Chen X, Song Y, Caballero B, Cheskin LJ (2008). Association between obesity and kidney disease; a systematic review and meta-analysis. Kidney Int.

[3] Brito PL, Fioretto P, Drummond K, Kim Y, Steffes MW, Basgen JM, Sisson-Ross S, Mauer M (1998). Proximal tubular basement membrane width in insulin-dependent diabetes mellitus. Kidney Int.

[4] Thomas MC, Burns WC, Cooper ME (2005). Tubular changes in early diabetic nephropathy. Adv Chronic Kidney Dis.

[5] Serafini T, Kennedy TE, Galko MJ, Mirzayan C, Jessel TM, Tessier-Lavigne M (1994). The netrins define a family of axon outgrowth-promoting proteins homologous to C. elegans UNC-6. Cell.

[6] Cirulli V, Yebra M (2007). Netrins: beyond the brain. Nat Rev Mol Cell Biol.

[7] Ly NP, Komatsuzaki K, Fraser IP, Tseng AA, Prodhan P, Moore KJ, Kinane TB (2005). Netrin-1 inhibits leucocyte migration in vitro and in vivo. Proc Natl Acad Sci U S A.

[8] Ramesh G (2012). Role of netrin-1 Beyond the Brain: From Biomarker of Tissue Injury to Therapy for Inflammatory Diseases. Recent Pat Biomark.

[9] Wang W, Reeves WB, Ramesh G (2008). Netrin-1 and kidney injury. I. Netrin-1 protects against ischemia-reperfusion injury of the kidney. Am J Physiol Renal Physiol.

[10] Ramesh G, Krawczeski CD, Woo JG, Wang Y, Devarajan P (2010). Urinary netrin-1 is an early predictive biomarker of acute kidney injury after cardiac surgery. Clin J Am Soc Nephrol.

[11] Reeves WB, Kwon O, Ramesh G (2008). Netrin-1 and kidney injury. II. Netrin-1 is an early biomarker of acute kidney injury. Am J Physiol Renal Physiol.

[12] Jayakumar C, Nauta FL, Bakker SC, Bilo H, Gansevoort RT, Johnson MH, Ramesh G (2014). Netrin-1, a urinary proximal tubular injury marker, is elevated early in the time course of human diabetes. J Nephrol.

[13] Neyzi O, Gönöz H, Furman A, Bundak R, Gökçay G, Darendeliler F, Baş F (2008). Weight, height, head circumference and body mass index references for Turkish children. Turk J Pediatr.

[14] National High Blood Pressure Education Program Working Group on High Blood Pressure in Children and Adolescents (2004). The fourth report on the diagnosis, evaluation, and treatment of high blood pressure in children and adolescents. Pediatrics.

[15] and Blood Institute (2011). Expert panel on integrated guidelines for cardiovascular health and risk reduction in children and adolescents: summary report. Pediatrics.

[16] Kurtoğlu S, Hatipoğlu N, Mazıcıoğlu M, Kendirici M, Keskin M, Kondolat M (2010). Insulin resistance in obese children and adolescents: HOMA-IR cut-off levels in the prepubertal and pubertal periods. J Clin Res Pediatr Endocrinol.

[17] (2007). KDOQI clinical practice guidelines and clinical practice recommendations for diabetes and chronic kidney disease. Am J Kidney Dis.

[18] Marshall WA, Tanner JM (1969). Variation in the pattern of pubertal changes in girls. Arch Dis Child.

[19] Marshall WA, Tanner JM (1970). Variation in the pattern of pubertal changes in boys. Arch Dis Child.

[20] Schwartz GJ, Munoz A, Schneider MF, Mak RH, Kaskel F, Warady BA, Furth SL (2009). New equations to estimate GFR in children with CKD. J Am Soc Nephrol.

[21] Steinke JM, Sinaiko AR, Kramer MS, Suissa S, Chavers BM, Mauer M, International Diabetic Nephopathy Study a (2005). The early natural history of nephropathy in Type 1 Diabetes: III. Predictors of 5-year urinary albumin excretion rate patterns in initially normoalbuminuric patients. Diabetes.

[22] Goknar N, Oktem F, Ozgen IT, Kucukkoc M, Demir AD, Cesur Y (2015). Determination of early urinary injury markers in obese children. Pediatr Nephrol.

[23] Savino A, Pelliccia P, Giannini C, Giorgis T, Cataldo I, Chiarelli F, Mohn A (2011). Implications for kidney disease in obese children and adolescents. Pediatr Nephrol.

[24] Sarafidis PA, Ruilope LM (2006). Insulin resistance, hyperinsulinemia, and renal injury: mechanisms and implications. Am J Nephrol.

[25] White JJ, Mohamed R, Jayakumar C, Ramesh G (2013). Tubular injury marker netrin-1 is elevated early in experimental diabetes. J Nephrol.

